# Age-Associated Genetic Variations in Breast Cancer: Somatic Mutations and Co-Mutations

**DOI:** 10.3390/biomedicines14030510

**Published:** 2026-02-25

**Authors:** Busra Ekinci, Seda Orenay-Boyacioglu, Ibrahim Halil Erdogdu, Olcay Boyacioglu, Merve Cirak-Balta, Nesibe Kahraman-Cetin, Ibrahim Meteoglu

**Affiliations:** 1 Department of Medical Pathology, School of Medicine, Aydin Adnan Menderes University, Efeler 09010, Aydin, Türkiye; busra.ekinci@adu.edu.tr (B.E.); ibrahim.halil.erdogdu@adu.edu.tr (I.H.E.); merve.cirak.balta@adu.edu.tr (M.C.-B.); nesibe.cetin@adu.edu.tr (N.K.-C.); imeteoglu@adu.edu.tr (I.M.); 2 Department of Medical Genetics, School of Medicine, Aydin Adnan Menderes University, Efeler 09010, Aydin, Türkiye; sorenay@adu.edu.tr; 3 Faculty of Engineering, Aydin Adnan Menderes University, Efeler 09010, Aydin, Türkiye

**Keywords:** breast cancer, geriatric, somatic mutations, co-mutations, *PIK3CA*, *TP53*, molecular subtypes, immunohistochemistry

## Abstract

**Background/Objectives:** Breast cancer (BCa) is a heterogeneous disease with molecular and genetic characteristics that significantly influence prognosis and treatment strategies. Age-related differences in tumor biology may impact therapeutic decisions; however, data on somatic mutation profiles in geriatric patients are limited. **Methods:** This retrospective study included 371 BCa patients (53 geriatric ≥ 65 years, 318 non-geriatric) whose clinicopathological and next-generation sequencing (NGS) data were analyzed. Immunohistochemical markers and molecular subtypes were assessed according to ASCO/CAP guidelines. Mutational profiles were obtained using the QIAseq Human BCa Panel (93 genes). **Results:** Among all patients, 1669 somatic mutations were detected, and 93.3% of patients harbored at least one mutation. Mutation prevalence was similar between geriatric (96.2%) and non-geriatric (92.8%) groups (*p* = 0.526), indicating that age did not significantly affect overall mutational burden. The most frequently mutated genes were *ATR*, *TP53*, *PIK3CA*, *PTEN*, *RAD50*, *BLM*, *NF1*, *AR*, *BRCA2*, and *KMT2C*. Notably, *PIK3CA* mutations were significantly more frequent in geriatric patients (28.3% vs. 23.2%, *p* = 0.0418). *TP53* mutations correlated with higher Ki-67 proliferation indices (*p* = 0.035), while *ATR* mutations were more common in HER2-enriched subtypes (*p* = 0.002). **Conclusions:** Our findings indicate that while the overall somatic mutational load in BCa does not differ significantly with age, specific molecular alterations—particularly the enrichment of *PIK3CA* mutations in elderly patients—underscore the importance of integrating genomic profiling into personalized treatment planning. This study represents the first comprehensive molecular characterization of geriatric BCa patients in Türkiye, providing valuable insights for age-specific genetic profiling, treatment optimization, and future multicenter translational studies.

## 1. Introduction

Breast cancer (BCa) remains a major cause of morbidity and mortality among women worldwide. The biological processes associated with aging introduce distinct clinical and molecular challenges, particularly in older patients, through complex interactions between senescence and tumor biology. Emerging evidence suggests that geriatric patients (≥65 years) and younger individuals with BCa differ not only in clinical presentation but also in tumor biology and somatic mutational patterns, underscoring the need for age-adapted and individualized therapeutic strategies. The molecular features of BCa—most notably hormone receptor status and genomic alterations—play a central role in guiding treatment decisions and shaping prognostic expectations [1,2].

Clinicopathological studies have consistently shown that tumors in geriatric patients are more frequently hormone receptor–positive, exhibit lower proliferative activity, and follow a relatively indolent clinical course. In contrast, younger patients more often present with biologically aggressive molecular subtypes and accelerated disease progression [1,2]. From a therapeutic perspective, advanced age is commonly associated with reduced tolerance to cytotoxic chemotherapy, largely due to increased comorbidity burden and polypharmacy. As a result, dose modifications and treatment interruptions occur more frequently in this population, potentially influencing progression-free and overall survival outcomes [3,4]. Conversely, endocrine therapies generally remain effective in hormone receptor–positive elderly patients; however, the use of targeted agents—such as PI3K inhibitors—may be limited by age-related toxicity concerns that complicate clinical decision-making [5]. Survival analyses from large cohort studies indicate that cancer-specific survival in geriatric patients may be comparable to that of younger individuals, whereas overall survival is often inferior, reflecting the cumulative impact of comorbid conditions and differences in treatment accessibility and tolerability [6,7].

Despite these clinically relevant distinctions, most genomic investigations in BCa have predominantly focused on young and middle-aged populations, leaving geriatric patients underrepresented in both clinical trials and molecular studies. The limited available data suggest that somatic mutational landscapes in elderly patients may differ substantially from those observed in younger cohorts, with certain prognostically significant genes—such as *PIK3CA*—exhibiting higher mutation frequencies with increasing age [5]. Although some reports have described a greater prevalence of *BRCA1/2* mutations in postmenopausal women, comprehensive and systematic data characterizing somatic mutation spectra and co-mutation patterns in geriatric BCa patients remain scarce on a global scale [6,7].

Importantly, while the indolent clinical phenotype observed in many geriatric BCa cases is well recognized, the underlying somatic genomic mechanisms driving these age-related differences are not yet fully understood. Age-associated increases in comorbidity burden, alterations in pharmacokinetics, and variability in treatment tolerance further emphasize the clinical importance of defining molecular profiles specific to older patients. In this context, comparative analyses of somatic mutation and co-mutation patterns between geriatric and non-geriatric BCa patients represent a critical step toward elucidating age-dependent biological differences and advancing precision oncology approaches. The present study aims to provide one of the first comprehensive evaluations of the somatic mutational landscape in geriatric BCa patients in Türkiye, thereby addressing a significant gap in the literature and contributing to the development of more personalized and inclusive treatment strategies.

## 2. Materials and Methods

### 2.1. Ethical Approval and Inclusion Criteria

This study was approved by the Institutional Non-Interventional Clinical Research Ethics Committee of Aydin Adnan Menderes University School of Medicine (2025/#198). The principles of the Declaration of Helsinki were adhered to throughout the study.

This retrospective study utilized data extracted from a prospectively established database. Medical records of all BCa patients who were followed at the Department of Oncology, Aydin Adnan Menderes University Faculty of Medicine, and referred to the Molecular Pathology Laboratory between 2018 and 2025 were reviewed. Patients were included in the study if they had available data on hormone receptor status, proliferative cell nuclear antigen (Ki-67) index, and next-generation sequencing (NGS) BCa panel results. Patients lacking any of these data were excluded from the analysis. A total of 371 patients met the inclusion criteria and were included in the final analyses. For each patient, demographic information, age group, tumor histopathology, ER, PR, HER2 status, Ki-67 index, and somatic mutation profiles were evaluated. Patients were categorized into two age-based groups: the geriatric group, comprising individuals aged ≥ 65 years, and the non-geriatric group, including patients aged 40–64 years. The ≥65-year threshold was selected because it has been widely used in international oncology literature and has been recommended by the International Society of Geriatric Oncology (SIOG) as the standard cut-off applied in clinical decision-making rather than biological age [8,9,10].

Immunohistochemical (IHC) staining, fluorescence in situ hybridization (FISH) analysis, molecular classification, and NGS panel analyses were performed in accordance with the protocols described by Erdogdu et al. (2023, 2024) [11,12].

### 2.2. IHC Staining and FISH Analysis

Hormone receptor status was evaluated in accordance with the American Society of Clinical Oncology/College of American Pathologists (ASCO/CAP) guidelines [5]. Tumor tissues were fixed in 10% formalin and embedded in paraffin blocks. Sections of 5 µm thickness were prepared, followed by dewaxing and hydration procedures. Endogenous peroxidase activity was blocked using 3% H_2_O_2_, and antigen retrieval was performed using the microwave method. After blocking with normal serum, the sections were incubated overnight at 4 °C with primary antibodies against ER, PR, HER2, and Ki-67. Visualization was achieved using biotinylated secondary antibodies and diaminobenzidine (DAB), followed by hematoxylin counterstaining, dehydration, and mounting. For ER and PR, nuclear staining in more than 1% of tumor cells was considered positive. HER2 scores were evaluated according to Dako criteria: scores of 0–1+ were considered negative, 3+ positive, and cases with a score of 2+ were confirmed by FISH. A Ki-67 labeling index of ≥14% was regarded as high expression.

*HER2* gene amplification was assessed using the Dako dual-color probe kit. Four-micrometer sections were deparaffinized and treated with sodium thiocyanate and protease, followed by hybridization with the probe for 12 h. Post-hybridization washing and DAPI counterstaining were performed, and slides were analyzed under a fluorescence microscope. Cases with a *HER2/CEP17* ratio ≥ 2 or an average *HER2* copy number ≥ 4 were considered positive.

### 2.3. Molecular Classification

Tumors were classified according to their histopathological and IHC features. Cases that were ER(+) and/or PR(+), HER2(−), and had a Ki-67 index < 20% were categorized as Luminal A. Tumors that were ER(+) and/or PR(+), HER2(−), with a Ki-67 ≥ 20% were classified as Luminal B-HER2(−). Cases that were ER(+) and/or PR(+), HER2(+) were classified as Luminal B-HER2(+). Tumors that were ER(−), PR(−), and HER2(+) were defined as HER2-positive, while those that were ER(−), PR(−), and HER2(−) were classified as triple-negative breast cancer.

### 2.4. NGS Panel Analysis

Genomic DNA was extracted from formalin-fixed paraffin-embedded (FFPE) tissue samples using the QIAamp DNA FFPE Tissue Kit (Qiagen, Hilden, Germany). DNA purity and concentration were assessed spectrophotometrically, and only samples with an OD260/OD280 ratio between 1.8 and 2.0 were included in the NGS analysis. Sequencing was performed on the Illumina MiSeq platform using the QIAseq Human BCa Panel (DHS-001Z, Qiagen, Hilden, Germany), which targets 93 genes (*ACVR1B*, *CDKN2A*, *GEN1*, *NCOR1*, *SEPT9*, *AKT1*, *CHEK2*, *HERC1*, *NEK2*, *SMAD4*, *APC*, *CSMD1*, *HOXB13*, *NF1*, *SMARCA4*, *AR*, *CTNNB1*, *IRAK4*, *PALB2*, *STK11*, *ATM*, *DIRAS3*, *ITCH*, *PALLD*, *SYNE1*, *ATR*, *EGFR*, *KMT2C*, *PBRM1*, *TGFB1*, *AXIN2*, *EP300*, *KRAS*, *PCGF2*, *TP53*, *BAP1*, *EPCAM*, *MAP2K4*, *PIK3CA*, *TRAF5*, *BARD1*, *ERBB2*, *MAP3K1*, *PIK3R1*, *VHL*, *BLM*, *ERBB3*, *MDM2*, *PMS1*, *WEE1*, *BMPR1A*, *ERCC4*, *MED12*, *PMS2*, *XRCC2*, *BRCA1*, *ESR1*, *MEN1*, *PPM1L*, *XRCC3*, *BRCA2*, *EXOC2*, *MLH1*, *PTEN*, *ZBED4*, *BRIP1*, *EXT2*, *MRE11A*, *PTGFR*, *CASP8*, *FAM175A*, *MSH2*, *RAD50*, *CBFB*, *FANCC*, *MSH6*, *RAD51*, *CCND1*, *FBXO32*, *MUC16*, *RAD51C*, *CDH1*, *FGFR1*, *MUTYH*, *RAD51D*, *CDK4*, *FGFR2*, *MYC*, *RB1*, *CDK6*, *GATA3*, *NBN*, *RET*) and includes 4831 primers. The panel covers all exon regions and exon–intron boundaries of the selected genes. The mean sequencing depth across target regions exceeded 500×, ensuring high sensitivity for the detection of somatic variants in FFPE-derived DNA. Only variants meeting the minimum coverage threshold of ≥100× were considered for downstream analyses. Variant calling was performed using Qiagen Clinical Insight Interpret (QCI™) software (v8.1.202021). A variant allele frequency cutoff of ≥5% was applied, in line with commonly accepted standards for somatic variant detection in targeted NGS panels. Variants below this threshold were excluded to minimize false-positive calls related to sequencing artifacts. All detected variants were classified according to the American College of Medical Genetics and Genomics (ACMG) guidelines [7]. Variants classified as pathogenic or likely pathogenic were included in the mutation and co-mutation analyses presented in the manuscript. Variants of uncertain significance (VUS) were systematically identified and reported by the QCI™ pipeline; however, VUS were excluded from statistical comparisons and frequency analyses to avoid overinterpretation of variants without established clinical or biological relevance. Benign and likely benign variants were also excluded from all analyses.

### 2.5. Statistical Analysis

All statistical analyses were performed using IBM SPSS Statistics version 25.0 (IBM Corp., Armonk, NY, USA), and a *p*-value ≤ 0.05 was considered statistically significant. Descriptive statistics were used to summarize the data, with categorical variables reported as frequencies and percentages, and continuous variables presented as medians with minimum and maximum values. Associations between categorical variables were evaluated using the Chi-square test or Fisher’s exact test, depending on data distribution and expected cell counts. In co-mutation analyses, Fisher’s exact test was applied when the expected cell count was <5, whereas the Chi-square test was used in all other cases. For tables involving multiple comparisons, Bonferroni correction was considered. Correlations between variables were assessed using Spearman’s rank-order correlation coefficient.

## 3. Results

A total of 371 patients with BCa were included in the study. The majority were female (n = 358, 96%), while 13 patients (4%) were male. Based on chronological age, 53 patients (14.3%) were classified as geriatric (≥65 years), and 318 patients (85.7%) as non-geriatric (<65 years). There was no statistically significant difference in sex distribution between the geriatric and non-geriatric groups (female: 96.2% vs. 96.5%; male: 3.8% vs. 3.5%; *p* = 0.68).

Histopathologically, invasive ductal carcinoma (IDC) was the most common subtype, identified in 294 patients (79.2%), followed by invasive lobular carcinoma (ILC) in 12 cases (3.2%). Other less frequent histological subtypes, including malignant epithelial tumor, invasive apocrine carcinoma, and solid papillary carcinoma, were collectively observed in 65 patients (17.5%). In the geriatric group, IDC was present in 27 patients (50.9%), ILC in 2 patients (3.8%), and other specific histologic types in 24 patients (45.3%). In contrast, within the non-geriatric group, IDC was found in 267 patients (84.0%), ILC in 10 patients (3.1%), and other histologic types in 41 patients (12.9%).

IHC analysis revealed ER positivity in 75.7% of cases and PR positivity in 66.8%. HER2 positivity (IHC 3+ or FISH-confirmed amplification) was observed in 22.9% of patients. Additionally, high Ki-67 proliferative index (≥20%) was detected in 45.8% of the cohort.

According to molecular subtyping, 32.3% of all cases were classified as Luminal A, 24.5% as Luminal B-HER2(−), 10.5% as Luminal B-HER2(+), 12.4% as HER2-positive (non-luminal), and 15.9% as triple-negative. In 4.4% of patients, the subtype could not be determined due to insufficient clinicopathological data. The most common subtype in the geriatric group was Luminal A (41.5%), whereas Luminal B-HER2(−) (26.4%) predominated in the non-geriatric group. The triple-negative subtype was more frequent in the non-geriatric group.

In the IHC analysis, the median expression levels of ER, PR, Ki-67, and P53 were comparable between the two groups. The median ER expression was 60% in both the geriatric and non-geriatric patients (Mann–Whitney U test, *p* = 0.618). Similarly, the median PR levels were 10% in both groups (*p* = 0.983). The median Ki-67 index was 20% in both the geriatric and non-geriatric groups (*p* = 0.519), while the median P53 expression was 10% in geriatric patients and 5% in non-geriatric patients (*p* = 0.926). None of these differences were statistically significant. *HER2*/FISH positivity was detected in 10 of 53 geriatric patients and in 81 of 318 non-geriatric patients (χ^2^ test, *p* = 0.389). E-cadherin positivity was observed in 18 of 53 geriatric and 139 of 318 non-geriatric patients (χ^2^ test, *p* = 0.238). No significant intergroup differences were observed for either biomarker (*p* > 0.05). Table 1 summarizes the demographic, histopathological, immunohistochemical, and molecular subtype characteristics of the entire BCa cohort, stratified into geriatric and non-geriatric groups. Comparisons between age groups were performed to evaluate age-related differences in tumor phenotype and biomarker distribution.

A total of 1669 mutations were detected in 371 patients, with at least one mutation identified in 346/371 (93.3%) cases. The most frequently mutated genes in the entire cohort were *ATR*, *TP53*, *PIK3CA*, *PTEN*, *RAD50*, *BLM*, *NF1*, *AR*, *BRCA2*, and *KMT2C* (Table 2).

**Figure 1:**
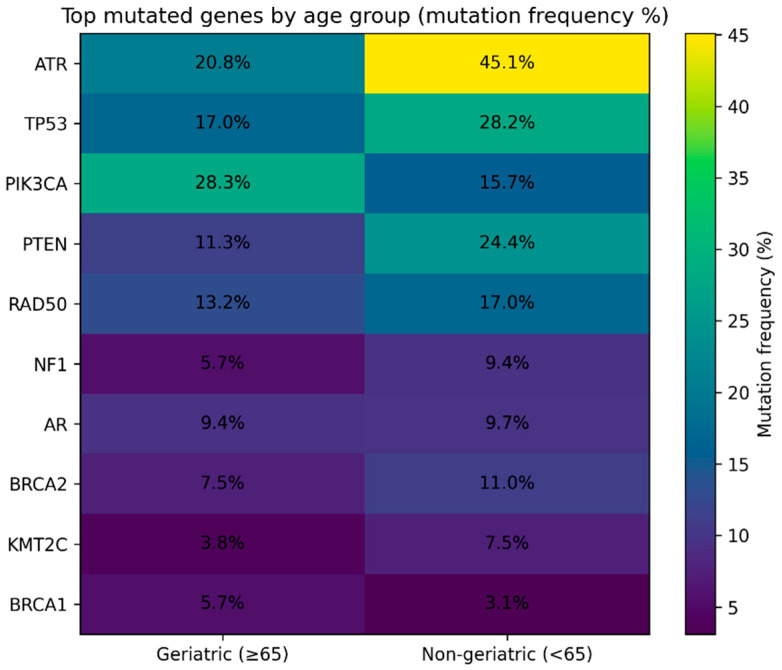
Age-specific mutation frequencies in BCa. Heatmap showing the mutation frequencies (%) of the most frequently mutated genes in BCa patients, stratified by age group [geriatric (≥65 years) vs. non-geriatric (<65 years)]. Color intensity represents mutation frequency, and values indicate the percentage of patients with at least one somatic mutation in each gene.

**Figure 2:**
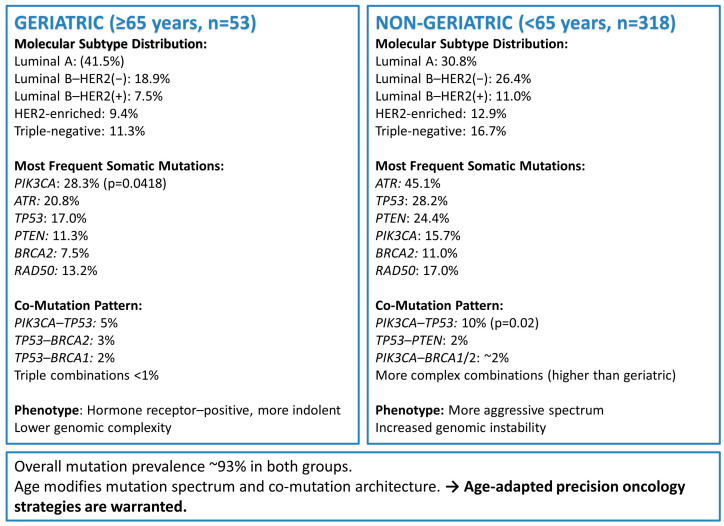
Schematic Summary of Age-Associated Molecular Differences in BCa. This schematic summary illustrates the age-associated molecular differences identified between geriatric (≥65 years, n = 53) and non-geriatric (<65 years, n = 318) BCa patients.

Among the geriatric patients, 51 of 53 (96.2%), and among the non-geriatric patients, 295 of 318 (92.8%) harbored at least one mutation. There was no statistically significant difference in the overall mutation frequency between the two groups (χ^2^ test, *p* = 0.526). The most frequently mutated genes in the geriatric cohort were *PIK3CA* (28.3%), *ATR* (20.8%), and *BRCA1* (5.7%), whereas in the non-geriatric group, *ATR* (45.1%), *TP53* (28.2%), *PTEN* (24.4%), and *PIK3CA* (23.2%) predominated (Figure 1). Analysis of the top 15 frequently mutated genes revealed that *PIK3CA* mutations were significantly more common in geriatric patients compared with non-geriatric patients (15/53 vs. 50/318, *p* = 0.0418, χ^2^ test). Differences in the frequency of other genes were not statistically significant (*p* > 0.05). Table 2 presents the frequency of the most commonly detected somatic gene mutations in geriatric and non-geriatric BCa patients. Mutation frequencies are reported as percentages, and statistical comparisons highlight age-associated differences in the somatic mutational landscape. Figure 2 summarizes the age-associated differences in molecular subtype distribution, somatic mutation frequencies, and co-mutation patterns between geriatric and non-geriatric breast cancer patients.

A significant association was identified between *TP53* mutation status and Ki-67 expression. The median Ki-67 index was 70% in *TP53*-mutant cases and 20% in *TP53* wild-type cases (Mann–Whitney U test, *p* = 0.035), suggesting a link between *TP53* mutations and high proliferative activity. When molecular subtypes were compared, ATR mutations showed a significant association (χ^2^, *p* = 0.002), being more prevalent in the HER2-enriched subtype (≈40% within this group). No significant correlations were observed between other common mutations (*PIK3CA*, *PTEN*, *BRCA2*, etc.) and ER, PR, or HER2 status (*p* > 0.05).

Across the entire cohort, the overall co-mutation rate ranged between 5% and 7%. Comparative analysis between geriatric and non-geriatric patients revealed recurrent co-occurrences of certain genes. The most frequent co-mutation was identified between *PIK3CA* and *TP53*, occurring more commonly in the non-geriatric group (10% vs. 5%, *p* = 0.02). Less frequent co-mutations were observed between *PIK3CA–BRCA1/2* and *TP53–BRCA1/2*. Differences in *BRAF–KRAS* and *PIK3CA–ERBB2* co-mutations were not statistically significant. More complex mutation combinations, such as *PIK3CA* + *TP53* + *BRCA1*, were rare (<1% in both groups). Overall, co-mutation profiling indicated a notable tendency for concurrent alterations in *PIK3CA* and *TP53* genes. Table 3 shows recurrent mutation and co-mutation patterns observed in the study cohort, stratified by age group. The analysis aims to identify age-specific co-occurring genomic alterations that may contribute to differences in tumor biology and clinical behavior between geriatric and non-geriatric patients.

## 4. Discussion

This study presents a comparative analysis of the clinicopathological characteristics and molecular profiles of geriatric (≥65 years) and non-geriatric (<65 years) BCa patients. Conducted on a cohort of 371 individuals, the findings reveal distinct molecular differences between age groups, most notably a significantly higher frequency of *PIK3CA* mutations in geriatric patients.

In the present cohort, comprising 371 BCa cases, the predominance of female patients aligns with the well-established epidemiological pattern of BCa being overwhelmingly more common in women, while male cases remain rare [13]. The absence of a significant difference in sex distribution between the geriatric and non-geriatric groups is consistent with previous studies [9] and indicates that both cohorts were balanced with respect to gender. This balance minimizes the potential confounding influence of sex on the observed molecular and clinicopathological differences.

Invasive ductal carcinoma was the most prevalent histopathological subtype in both age groups, which is in accordance with established pathological distributions [14]. However, the markedly higher proportion of special-type carcinomas in the geriatric group is a noteworthy finding, suggesting that histopathological diversity may increase with advancing age.

In this study, molecular subtype and IHC biomarker analyses revealed that Luminal A was the most frequent subtype in geriatric patients, whereas Luminal B–HER2(−) was the predominant subtype in non-geriatric patients. These findings are consistent with previously published studies [15]. The higher frequency of hormone receptor–positive and less aggressive tumors in elderly individuals reflects age-related changes in tumor biology. This phenomenon may be explained by prolonged lifetime exposure to estrogen as well as immunosenescence, which collectively favor the development of more indolent tumor phenotypes [16]. Conversely, the higher proportion of triple-negative BCa observed in non-geriatric patients supports the notion of a more aggressive tumor biology in younger individuals. This observation may be linked to the greater prevalence of *BRCA1/2* germline mutations and homologous recombination deficiencies reported in younger-onset cases [17].

Regarding IHC markers, no statistically significant differences were observed between the groups in ER, PR, Ki-67, or P53 expression levels, suggesting that these biomarkers do not vary substantially with age and maintain similar prognostic relevance across patient populations. Previous studies have likewise reported that the expression and interrelationships of Ki-67, HER2, P53, ER, and PR do not differ significantly between age categories [18]. Similarly, the absence of age-related differences in HER2 and E-cadherin positivity aligns with the literature [19], indicating that despite chronological age differences, these biomarkers exhibit comparable prognostic and predictive behavior in both younger and older patients.

A total of 1669 mutations were identified in this study, and at least one mutation was detected in 93.3% of the patients. There was no statistically significant difference in mutation prevalence between the two groups. This finding indicates that age does not have a significant impact on the overall mutational burden. Similarly, previous studies have also reported no substantial difference in total somatic mutation load between younger and older BCa patients. Mealey et al. (2020) compared BCa patients diagnosed at a young age (<40 years) with those diagnosed at an older age (>60 years) and found that the overall mutation burden did not differ significantly between the age groups [20]. Likewise, Qing et al. (2022) reported no marked difference in mutation load among estrogen receptor-positive BCa patients across different age groups [21]. Moreover, in a large-scale study published by Selenica et al. (2025), although mutations in DNA damage repair genes were observed at slightly lower frequencies in certain subtypes of older BCa patients, no statistically significant difference was found in overall mutational burden [5].

One of the key findings of this study is that *PIK3CA* mutations were significantly more frequent in the geriatric group compared to the non-geriatric group. This result is highly consistent with previous reports in the literature. In a metastatic BCa cohort, Gupta et al. (2023) demonstrated that *PIK3CA* mutations occurred at higher frequencies among patients over 70 years of age compared with those aged 50–69 or under 50 (44.4% vs. 31.6% vs. 26.7%, respectively) [15]. There are several possible explanations for the higher prevalence of *PIK3CA* mutations in older patients: (i) Cumulative mutational burden: The decline in DNA damage repair efficiency and prolonged exposure to carcinogens with aging may lead to an increased overall mutation load [22]. (ii) Association with HR(+) tumors: *PIK3CA* mutations are particularly enriched in HR(+)/HER2(–) subtypes, which are more common among elderly patients [23]. (iii) Clonal evolution: With advancing age, tumor cells may undergo prolonged clonal evolution, allowing *PIK3CA*-mutant clones to gain a selective growth advantage [24].

In our study, the presence of *TP53* mutations was found to be significantly associated with the Ki-67 proliferation index. This finding can be explained by the critical role of *TP53* in cell cycle regulation. The *TP53* gene product, often referred to as the “guardian of the genome,” functions to halt the cell cycle in response to DNA damage, initiating repair processes or inducing apoptosis in cells with irreparable damage [25]. When *TP53* is mutated, several key cellular control mechanisms are disrupted, including loss of cell cycle checkpoints, impairment of apoptotic pathways, uncontrolled cellular proliferation, and increased genomic instability. These mechanisms collectively explain the elevated Ki-67 index observed in *TP53*-mutant tumors, which is often correlated with a more aggressive tumor phenotype [26].

The significant association between *ATR* mutations and molecular subtypes, particularly their higher prevalence in the HER2-enriched subgroup, is a noteworthy finding. The *ATR* gene plays a crucial role in the DNA damage response, especially under conditions of replication stress [27]. The increased replication stress commonly observed in HER2(+) tumors may lead to enhanced activation of the ATR pathway as an adaptive cellular mechanism, which could explain the higher mutation frequency in this molecular subtype.

Another notable finding in our study is that *PIK3CA–TP53* co-mutations were the most frequent co-occurring mutations, and their prevalence was significantly higher in the non-geriatric group compared to the geriatric group. The *PIK3CA–TP53* co-mutation represents the simultaneous disruption of distinct but complementary signaling pathways: *PIK3CA* mutations activate the PI3K/AKT/mTOR pathway, promoting cell growth and survival, while *TP53* mutations lead to the loss of cell cycle control and apoptosis regulation. This dual alteration may enhance proliferative capacity and genomic instability, potentially contributing to more aggressive tumor behavior and therapeutic resistance compared to single-gene alterations. In contrast, the lower co-mutation frequency observed in geriatric patients may be consistent with their relatively less aggressive tumor phenotype. Thus, co-mutation profiling may provide additional mechanistic and prognostic insight beyond individual mutations and support age-adapted precision oncology strategies [28]. Consistent with our findings, previous studies have reported that *PIK3CA–TP53* co-mutant tumors exhibit distinct clinicopathological characteristics and may demonstrate altered therapeutic responses compared to single-mutant tumors [29].

When the somatic alterations identified in this study are evaluated in terms of their biological and clinical relevance, clear age-related differences emerge among well-established driver mutations. Alterations in key genes involved in BCa pathogenesis, particularly *TP53*, *PIK3CA*, and *PTEN*, influence tumor behavior through disruption of cell cycle regulation, DNA damage response mechanisms, and activation of the PI3K/AKT/mTOR signaling cascade. The increased prevalence of *PIK3CA* mutations in geriatric patients suggests the presence of an age-associated molecular pattern that may contribute to the more indolent clinical phenotype and favorable response to endocrine therapy commonly observed in this population. At the same time, this finding carries important therapeutic implications, as activating *PIK3CA* mutations represent an established predictive biomarker for PI3K inhibition in HR-positive/HER2-negative BCa. In the phase III SOLAR-1 trial, alpelisib combined with fulvestrant significantly improved progression-free survival in patients harboring *PIK3CA* mutations [30]. However, in elderly patients, careful consideration of comorbidities, particularly diabetes and cardiovascular disease, and the risk of alpelisib-induced hyperglycemia is essential, given the higher likelihood of polypharmacy and drug–drug interactions in this population [31]. Therefore, *PIK3CA*-targeted therapies in geriatric patients should be implemented only after a thorough risk–benefit assessment [32].

In contrast, the higher frequency of mutations affecting DNA damage response genes, such as *TP53* and *ATR*, in non-geriatric patients is consistent with a molecular profile characterized by increased genomic instability, enhanced proliferative capacity, and more aggressive tumor biology. Although direct *TP53*-targeted therapies are not yet established, these molecular features may support the exploration of DNA damage response–targeted strategies, including *ATR* inhibition [22,24,25,27,29]. Integrating age-specific mutation patterns with clinical parameters may therefore enhance individualized treatment selection in routine practice.

Beyond single-gene alterations, the biological significance of co-mutation patterns further refines this age-related molecular stratification. The most frequent co-mutation observed in our cohort, *PIK3CA–TP53*, particularly in non-geriatric patients, represents the concurrent activation of the PI3K/AKT/mTOR pathway and loss of *TP53*-mediated cell cycle control. *PIK3CA*-driven signaling promotes tumor growth and survival [26], while *TP53* alterations are associated with genomic instability and adverse prognosis [25]. The coexistence of these alterations may therefore amplify proliferative capacity and therapeutic resistance compared to single-gene mutations. Conversely, the lower co-mutation frequency observed in geriatric patients may align with their relatively less aggressive tumor phenotype. Thus, co-mutation profiling provides additional mechanistic and potential prognostic insight beyond individual mutations and may support age-adapted precision oncology strategies.

Low-frequency variants and those with uncertain functional significance were interpreted cautiously as exploratory findings. Accordingly, the present discussion focuses primarily on well-characterized driver mutations supported by robust literature evidence, while putative passenger variants are considered reflections of age-related genomic heterogeneity rather than direct determinants of tumor behavior. This approach strengthens the biological interpretability of our findings and ensures that clinical implications remain centered on molecular alterations with established pathogenic and translational relevance.

Our results collectively suggest that age-specific molecular profiles should be integrated into treatment decision-making for BCa patients. In geriatric patients, the predominance of Luminal A tumors supports prioritization of endocrine therapy, and routine genomic testing may help identify candidates for targeted PI3K inhibition. Given the generally less aggressive tumor biology observed in this group, de-escalated treatment strategies may also be appropriate. In contrast, in non-geriatric patients, the higher incidence of triple-negative BCa and the increased prevalence of *PIK3CA–TP53* co-mutations highlight the potential need for more intensive systemic approaches and combinatorial strategies targeting multiple oncogenic pathways simultaneously.

Limitations: This study has several limitations. It was conducted at a single center, and although a total of 371 patients were included, the geriatric subgroup comprised only 53 individuals, resulting in an imbalance between age cohorts. This disproportion in sample sizes may limit the generalizability of the findings and reduce statistical power, particularly in the analysis of low-frequency mutations and co-mutation patterns, thereby increasing the risk of Type II error. While the age distribution reflects real-world clinical practice in patients undergoing NGS testing, the unequal cohort sizes may have introduced statistical asymmetry in intergroup comparisons. Therefore, future multicenter studies with larger and more balanced cohorts are required to confirm the age-associated mutation patterns identified in this study. In addition, due to the retrospective design of the study, a prospective power analysis could not be performed, and there is a possibility of heterogeneity or incompleteness in the clinical data. The absence of survival and recurrence data in the available database limits the ability to correlate the detected mutations with clinical outcomes. Finally, the absence of germline mutation analysis restricted the ability to distinguish between somatic and inherited variants, which may have limited the depth of insight into the genetic landscape of age-related BCa.

## 5. Conclusions

This study demonstrates that the molecular profiles of BCa differ according to patient age, highlighting important biological distinctions between geriatric and non-geriatric populations. The significantly higher frequency of *PIK3CA* mutations in geriatric patients underscores the need to develop age-specific molecularly targeted therapeutic strategies. Moreover, the strong association between *TP53* mutations and Ki-67 index, along with the co-occurrence of *PIK3CA–TP53* mutations, emphasizes the clinical relevance of combined biomarker approaches in understanding tumor aggressiveness and treatment response. Collectively, these findings support the notion that age should be considered a critical variable in precision oncology, and they advocate for the advancement of molecular profiling–based treatment strategies in geriatric BCa management.

## Figures and Tables

**Table 1:** Clinicopathological characteristics of BCa patients stratified by age.

	Variable	Total n (%)	Geriatric n (%)	Non-Geriatric n (%)	*p*-Value
Gender	Female	358 (96.5)	51 (96.2)	307 (96.5)	0.68
	Male	13 (3.5)	2 (3.8)	11 (3.5)	0.68
Histopathological Type	IDC	294 (79.2)	27 (50.9)	267 (84.0)	<0.001
	ILC	12 (3.2)	2 (3.8)	10 (3.1)	0.81
	Other special types	65 (17.5)	24 (45.3)	41 (12.9)	<0.001
Hormone Receptor Status	ER(+)	281 (75.7)	40 (75.5)	241 (75.8)	0.96
	PR(+)	248 (66.8)	35 (66.0)	213 (67.0)	0.89
	HER2(+) (IHC 3+ or FISH+)	85 (22.9)	10 (18.9)	75 (23.6)	0.39
	Ki-67 ≥ 20%	170 (45.8)	22 (41.5)	148 (46.5)	0.53
Molecular Subtype	Luminal A	120 (32.3)	22 (41.5)	98 (30.8)	0.12
	Luminal B-HER2(−)	91 (24.5)	10 (18.9)	81 (26.4)	0.24
	Luminal B-HER2(+)	39 (10.5)	4 (7.5)	35 (11.0)	0.48
	HER2-enriched (non-luminal)	46 (12.4)	5 (9.4)	41 (12.9)	0.52
	Triple negative	59 (15.9)	6 (11.3)	53 (16.7)	0.33
Immunohistochemical expression levels	ER expression Median % (min–max)	60 (0–100)		60 (0–100)	0.618
	PR expression Median % (min–max)	10 (0–90)		10 (0–95)	0.983
	Ki-67 index Median % (min–max)	20 (5–90)		20 (5–95)	0.519
	p53 expression Median % (min–max)	10 (0–90)		5 (0–95)	0.926

**Table 2:** Distribution of the most frequently mutated genes according to age group.

Gene	Mutation Role	Function/Pathway	Geriatric n (%)	Non-Geriatric n (%)	Total n (%)	*p*-Value
*PIK3CA*	Driver	PI3K/AKT/mTOR	15 (28.3)	50 (15.7)	65 (17.5)	0.0418 *
*TP53*	Driver	Tumor suppressor	9 (17.0)	90 (28.2)	99 (26.7)	0.09
*PTEN*	Driver	PI3K pathway inhibitor	6 (11.3)	78 (24.4)	84 (22.6)	0.07
*ATR*	Driver	DNA damage response	11 (20.8)	143 (45.1)	154 (41.5)	0.002 *
*BRCA1*	Driver	Homologous recombination	3 (5.7)	10 (3.1)	13 (3.5)	0.28
*BRCA2*	Driver	Homologous recombination	4 (7.5)	35 (11.0)	39 (10.5)	0.45
*RAD50*	Likely driver	DNA repair	7 (13.2)	54 (17.0)	61 (16.4)	0.52
*NF1*	Likely driver	RAS signaling	3 (5.7)	30 (9.4)	33 (8.9)	0.38
*KMT2C*	Epigenetic driver	Epigenetic regulation	2 (3.8)	24 (7.5)	26 (7.0)	0.39
*AR*	Context-dependent driver	Hormone receptor	5 (9.4)	31 (9.7)	36 (9.7)	0.94

* Statistical significance *p* < 0.05.

**Table 3:** Age-related mutation and co-mutation patterns in BCa.

Co-Mutations	Geriatric (%)	Non-Geriatric (%)	Total (%)	*p*-Value
*PIK3CA + TP53*	5	10	8	0.02 *
*PIK3CA + BRCA1*	1	2	1.5	0.35
*PIK3CA + BRCA2*	1	1	1	0.88
*TP53 + BRCA1*	2	3	2.5	0.50
*TP53 + BRCA2*	3	1	2	0.12
*BRCA1 + BRCA2*	0	1	0.5	0.42
*PIK3CA + TP53 + BRCA1*	0.5	1	0.8	0.55
*PIK3CA + TP53 + BRCA2*	0.5	0.5	0.5	0.99
*TP53 + PTEN*	1	2	1.5	0.40
*PIK3CA + AKT1*	0.5	1	0.8	0.60
*CDH1 + TP53*	0.5	1	0.8	0.55

* Statistical significance *p* < 0.05.

## Data Availability

The data presented in this study are available on request from the corresponding author due to privacy and ethical restrictions. The data were obtained retrospectively from patient medical records, and individual patient information cannot be shared publicly to protect confidentiality.

## Abbreviations

The following abbreviations are used in this manuscript:

ACMG	American College of Medical Genetics and Genomics
ASCO/CAP	American Society of Clinical Oncology/College of American Pathologists
BCa	Breast cancer
DAB	Diaminobenzidine
ER	Estrogen receptor
FFPE	Formalin-fixed paraffin-embedded
FISH	Fluorescence in situ hybridization
HER2	Human epidermal growth factor receptor 2
IDC	Invasive ductal carcinoma
IHC	Immunohistochemical
ILC	Invasive lobular carcinoma
NGS	Next-generation sequencing
PR	Progesterone receptor
QCI	Qiagen Clinical Insight Interpret
SIOG	International Society of Geriatric Oncology
VUS	Variants of uncertain significance
